# A Panel of Overexpressed Proteins for Prognosis in Esophageal Squamous Cell Carcinoma

**DOI:** 10.1371/journal.pone.0111045

**Published:** 2014-10-22

**Authors:** Li Shang, Hui-Juan Liu, Jia-Jie Hao, Yan-Yi Jiang, Feng Shi, Yu Zhang, Yan Cai, Xin Xu, Xue-Mei Jia, Qi-Min Zhan, Ming-Rong Wang

**Affiliations:** 1 State Key Laboratory of Molecular Oncology, Cancer Institute (Hospital), Peking Union Medical College and Chinese Academy of Medical Sciences, Beijing, China; 2 Department of Histology and Embryology, Anhui Medical University, Hefei, China; The University of Hong Kong, Hong Kong

## Abstract

Esophageal squamous cell carcinoma (ESCC) is a common cancer with poor prognosis. In order to identify useful biomarkers for accurately classifying prognostic risks for ESCC patients, we examined the expression of six proteins by immunohistochemistry (IHC) in 590 paraffin-embedded ESCC samples. The candidate proteins include p53, EGFR, c-KIT, TIMP1 and PI3K-p110α reported to be altered in ESCC tissues as well as another important component of PI3K, PI3K-p85α. Of the six proteins tested, p53, EGFR, c-KIT, TIMP1 and PI3K-p85α were detected with high expression in 43.0%, 36.6%, 55.9%, 70.7% and 57.1% of tumors, respectively. Significant associations were found between high expression of PI3K-p85α, EGFR and p53 and poor prognosis (*P* = 0.00111; 0.00001; 0.00426). Applying these three proteins as an IHC panel could divide patients into different subgroups (*P*<0.000001). Multivariate cox regression analysis indicated that the three-protein panel was an independent prognostic factor with very high statistical significance (HR = 2.090, 95% CI: 1.621–2.696, *P* = 0.00000001). The data suggest that the three-protein panel of PI3K-p85α, EGFR and p53 is an important candidate biomarker for the prognosis of patients with ESCC.

## Introduction

Esophageal squamous cell carcinoma (ESCC) is the predominant histological type of esophageal carcinoma in the “Asian esophageal cancer belt”. Although clinical treatment technologies have been developed in recent years, the prognosis of esophageal carcinoma is dismal [1]. Patient-specific tumor biomarkers correlated with prognosis could supplement current clinicopathologic parameters for risk stratification of ESCCs, which would be beneficial to clinical intervention and prolongation of patient survival.

The molecular genetic background of ESCC has been widely studied, and massive data focus on the change of proteins owing to their important implication of final executors of the cell activity and function. Alteration of expression level, molecular weight, subcellular localization, and post-translational modifications of proteins have been implicated in the tumorigenesis and development processes of ESCC [2], [3]. Researches on protein alterations in ESCC, especially those highly overexpressed, may have potentials to divide patients into different prognostic groups.

P53 was the most common protein with abnormality found in ESCC, and mutated p53 protein functionally promoted cell invasion and metastasis [4]. Epidermal growth factor receptor (EGFR), taking part in cellular differentiation and proliferation, was up-regulated in ESCC tissues [5], [6]. PI3K belongs to a family of lipid kinases that play crucial roles in various cellular processes. It is composed of a 110 kDa catalytic subunit (p110α, encoded by *PIK3CA* located at 3q26.3) and an 85 kDa regulatory subunit (p85α, encoded by *PIK3R1* located at 5q13.1) [7]. *PIK3CA* was amplified in ESCC [8], and the expression of PIK3CA mRNA and protein had been found to be associated with lymph node metastasis [9], [10]. Abnormal expression of PI3K-p85α protein had been observed in colon tumor tissues [11]. However, it remained undefined whether PI3K-p85α protein was altered in ESCC tissues.

Tyrosine kinase receptor c-KIT plays an important part in regulating cell survival, migration and proliferation [12]. It was overexpressed in many cancers, such as small cell lung carcinoma [13], breast cancer [14], epithelial ovarian tumors [15] and ESCC [16], [17]. Tissue inhibitors of matrix metalloproteinases 1 (TIMP1), as a negative regulator of Matrix metalloproteinases (MMPs) activity, plays a key role in maintaining the balance between extracellular matrix (ECM) deposition and degradation in different physiological processes. The implication of TIMP-1 in ESCC development, progression and formation of metastases had been most extensively characterized and best recognized [18].

In this study, immunohistochemistry (IHC) was performed to examine the expression changes of the above six candidate proteins in 590 paraffin-embedded tissue samples from ESCC patients with radical resection. Furthermore, we investigated clinical correlations of the protein alterations in order to provide a potential IHC panel for the prognosis of ESCC patients.

## Materials and Methods

### Ethics statement

This study was approved by the Ethics Committee/Institutional Review Board of the Cancer Institute (Hospital), PUMC/CAMS (No. 12-097/631).

### Patients and samples

590 surgically resected ESCC and morphologically normal operative margin tissues were collected between 1998 and 2009, in which 325 were from Cancer Hospital, CAMS/PUMC, Beijing, and 265 from Lin City People's Hospital, Henan, China. Every patient signed separate informed consent forms for sampling and molecular analysis. All the operative samples were residual specimens after diagnostic sampling. Tissues were routinely formalin-fixed and paraffin-embedded.

### Sample preparation and immunohistochemistry

Tissue microarrays (TMA) were constructed as described previously. For each case, tumor tissue was in triplicate and morphologically normal operative margin in duplicate as control. The resulting blocks were cut into 4-µm sections to prepare for immunohistochemistry (IHC) in accordance with a previously described protocol [19], [20]. The slides were deparaffinized, rehydrated, immersed in 3% hydrogen peroxide solution for 10 min, heated in citrate buffer (pH 6.0) for 25 min at 95°C, and cooled for 60 min at room temperature. Between each incubation step, the slides were washed with PBS (pH 7.4). Then the slides were incubated separately with anti-PI3-Kinase (PI3K) p85α mouse monoclonal antibody (1∶200 dilution, Clone: 4/PI3K-Kinase, BD Biosciences, California USA), anti-PIK3CA rabbit monoclonal antibody (1∶100 dilution, Clone: C73F8, Cell Signaling, Danvers, MA), anti-Epidermal Growth Factor Receptor (EGFR) mouse monoclonal antibody (1∶150 dilution, Clone: 31G7, invitrogen, Camarillo, CA), anti-p53 mouse monoclonal antibody (1∶150 dilution, Clone: DO-1, MBL, Nagoya, Japan), anti-Tissue inhibitors of matrix metalloproteinases 1 (TIMP1) rabbit polyclonal antibody (1∶100 dilution, Proteintech Group Inc, Chicago, USA), and anti-c-KIT rabbit polyclonal antibody (1∶600 dilution, Proteintech Group Inc, Chicago, USA) overnight at 4°C. Immunostaining was performed using the PV-9000 Polymer Detection System with diaminobenzidine (DAB) according to manufacturer recommendations (GBI, USA) and subsequently counterstained with hematoxylin. Slides with no primary antibodies added served as negative controls.

### Immunohistochemistry assessment

The results of the immunohistochemical staining were scored blindly with no information on the clinical data provided.

PI3K-p110α, TIMP1 and c-KIT protein expression were determined based on staining intensity: 0 (no staining), 1 (weak staining), 2 (moderate staining), and 3 (strong staining).

p53 protein expression was determined based on the percentage of immunoreactive cells, which was graded as 0 (no staining), 1 (<10%), 2 (10%–50%), and 3 (>50%).

PI3K-p85α and EGFR protein expression were determined based on staining intensity and the percentage of immunoreactive cells. The staining intensity was rated as 0 (no staining), 1 (weak staining), 2 (moderate staining), and 3 (strong staining). The percentage of immunoreactive cells was graded as 0 (no staining), 1 (<10%), 2 (10%–25%), 3 (26%–50%), and 4 (>50%). Tissue IHC score were calculated by multiplying the intensity and the percentage of positive tumor cells.

All cases were divided into two groups, a high group (score range: PI3K-p85α>3; PI3K-p110α>1, EGFR>2.2; p53>1, c-KIT>1, TIMP1>1) and a low group (score range: PI3K-p85α≤3; PI3K-p110α≤1, EGFR≤2.2; p53≤1, c-KIT≤1, TIMP1≤1). IHC assessment and imaging of TMAs were performed using a Leica DM2000 microscope equipped with Leica DFC Cameras-Image Acquisition System (software V3.5.0, Switzerland).

### Statistical analysis

All analyses were performed using the SPSS software program (SPSS Standard version 17.0, Chicago, IL). Comparing of the protein expressions between ESCC and adjacent normal tissues was performed using Paired t-test analysis. To assess the correlation of protein expressions with clinicopathologic parameters, χ2 test was used. For survival analyses, Kaplan-Meier curves were plotted by the Log-rank test. The clinical end point in the study was overall survival (OS), defined as time from surgery to death from ESCC or last contact. The data of patients alive at the end of the study were censored. Multivariate cox proportional hazards regression analysis was carried out to indentify the independent factors with a significant impact on patient survival. A difference was considered significant if the *P* value was less than 0.05.

## Results

### Protein expression in ESCC and adjacent normal esophageal tissues

**Figure 1:**
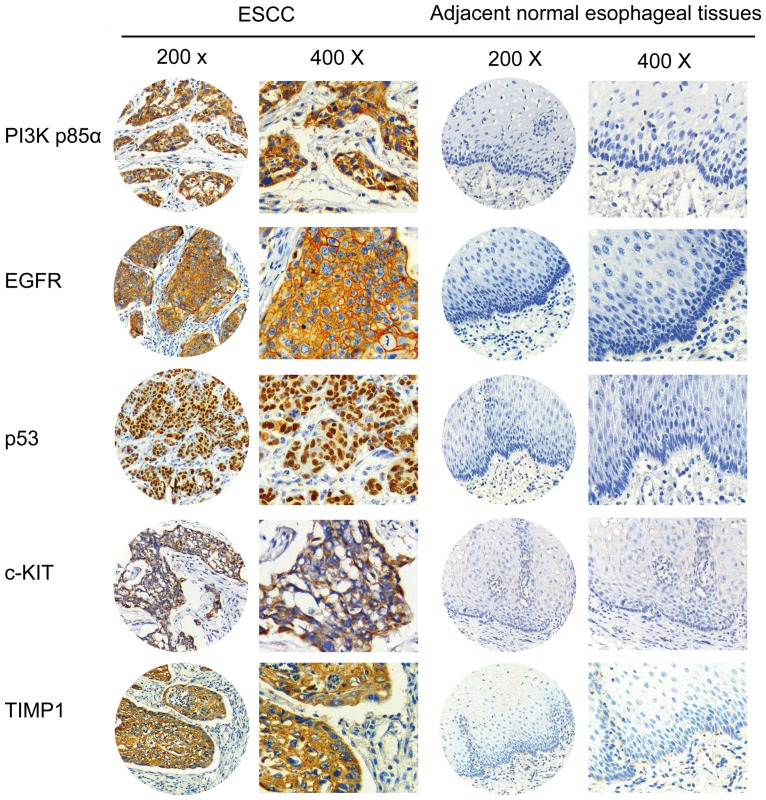
Representative IHC images of PI3K-p85α, EGFR, p53, c-KIT and TIMP1. IHC results reveal that these proteins are highly expressed in ESCC tumors, whereas a low/no expression in adjacent normal tissues. IHC, immunohistochemistry; ESCC, esophageal squamous cell carcinoma; PI3K, phosphatidylinositol 3-kinases; EGFR, epidermal growth factor receptor; TIMP1, TIMP metallopeptidase inhibitor 1. Original magnification: 200 × and 400 ×.

**Figure 2:**
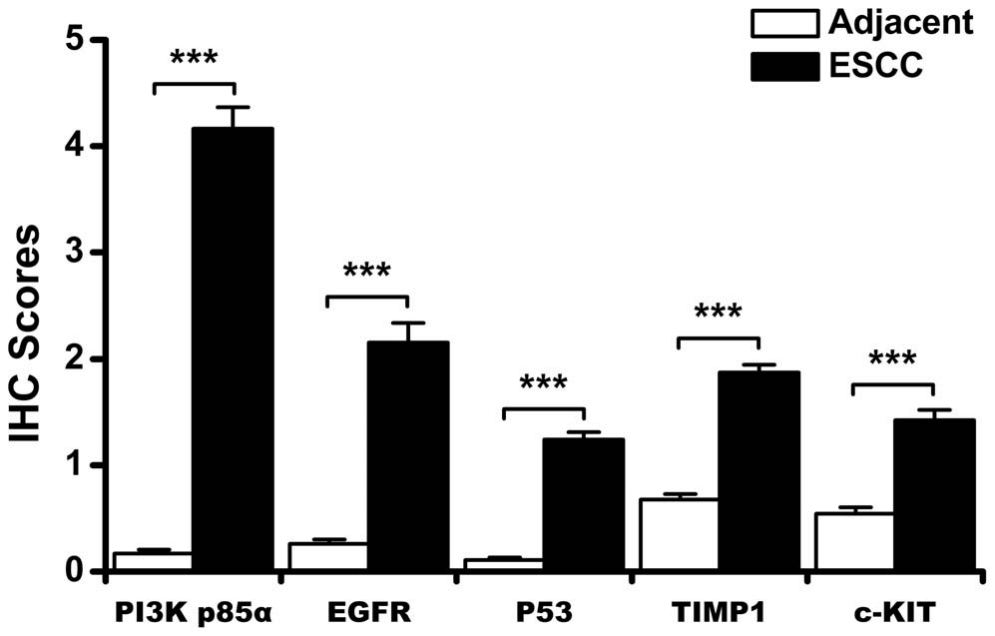
Significant differences of protein expression between ESCC and adjacent normal tissues (Paired Samples t Test). High expression of proteins in ESCC tumors (black bar graph), and low or no expression of proteins in adjacent normal tissues (white bar graph). Black horizontal lines are means, and error bars are SEs. *: *P*<0.05. **: *P*<0.01. ***: *P*<0.001. ESCC, esophageal squamous cell carcinoma; PI3K, phosphatidylinositol 3-kinases; EGFR, epidermal growth factor receptor; TIMP1, TIMP metallopeptidase inhibitor 1.

High expressions of candidate proteins PI3K-p85α, EGFR, p53, TIMP1 and c-KIT were detected in 57.1% (303/531), 36.6% (185/506), 43.0% (232/539), 70.7% (246/348) and 55.9% (104/186) of tumors, but in normal operative margin, 2.7% (10/368), 3.5% (13/372), 2.9% (11/382), 16.4% (41/250) and 9.6% (13/135), respectively (**Figure 1–2**). For the expression of PI3K-p110α, however, no significant differences were found between ESCC and adjacent normal epithelial tissues.

### Relationships between protein expression and clinicopathologic features

The high expressions of EGFR, p53 and TIMP1 were associated with macroscopic types (*P* = 0.002; 0.036; 0.00015). Higher EGFR and TIMP1 were observed more frequently in the carcinomas of upper thoracic segment esophagus (*P* = 0.032; 0.00046). A significant correlation was found between overexpression of EGFR, TIMP1 or c-KIT and pT (*P* = 2×10^−7^; 1×10^−10^; 0.00038). However, PI3K-p85α overexpression was not significantly correlated with clinicopathologic features (**Table 1**).

**Table 1:** Relationship between high expression of proteins and clinicopathologic parameters.

Clinical features	PI3K-p85α (%)	EGFR (%)	p53 (%)	TIMP1 (%)	c-KIT (%)
**Age at surgery, years**					
≤60	150 (56.8)	96 (39.3)	109 (40.5)	115 (68.0)	48 (53.3)
>60	153 (57.3)	89 (34.0)	123 (45.6)	131 (73.2)	56 (58.3)
* P*	0.910	0.210	0.238	0.293	0.492
**Sex**					
Female	80 (56.3)	50 (36.2)	65 (44.5)	71 (76.3)	30 (65.2)
Male	223 (57.3)	135 (36.7)	167 (42.5)	175 (68.6)	74 (52.9)
* P*	0.839	0.925	0.673	0.162	0.143
**Macroscopic types**					
Medullary	120 (56.6)	88 (45.8)	99 (46.0)	50 (52.6)	27 (47.4)
Ulcerative	69 (60.5)	30 (25.6)	41 (35.7)	83 (78.3)	35 (59.3)
Fungating	50 (56.8)	37 (45.7)	36 (39.1)	17 (45.9)	11 (44.0)
Unknown	4 (40.0)	5 (50.0)	1 (10.0)	3 (60.0)	2 (66.7)
* P*	0.627	0.002	0.036	0.00015	0.445
**Tumor location**					
Upper	37 (56.1)	30 (46.9)	28 (43.1)	41 (85.4)	21 (75.0)
Middle	185 (57.3)	102 (32.5)	137 (41.8)	161 (73.2)	59 (53.2)
Lower	78 (56.9)	52 (42.3)	67 (47.5)	42 (54.5)	23 (51.1)
* P*	0.983	0.032	0.514	0.00046	0.086
**Tumor size, cm**					
≤5	149 (56.4)	86 (34.0)	126 (46.5)	126 (71.6)	51 (56.0)
>5	144 (57.1)	94 (39.5)	102 (40.6)	111 (68.5)	48 (55.2)
* P*	0.872	0.206	0.178	0.538	0.907
**Histology grade**					
Good (G1)	88 (64.7)	41 (31.8)	60 (44.8)	68 (75.6)	26 (57.8)
Moderate (G2)	162 (55.1)	109 (38.7)	131 (43.0)	147 (72.4)	59 (54.6)
Poor (G3)	51 (53.7)	31 (34.4)	38 (40.4)	31 (58.5)	17 (54.8)
* P*	0.126	0.378	0.808	0.076	0.936
**pT**					
T1/T2	144 (57.4)	62 (25.1)	100 (40.3)	181 (83.0)	69 (67.6)
T3/T4	159 (56.8)	123 (47.5)	132 (45.4)	65 (50.0)	35 (41.7)
* P*	0.892	2×10^−7^	0.239	1×10^−10^	0.00038
**pN**					
N0	169 (60.1)	94 (33.7)	115 (39.5)	143 (73.3)	61 (59.2)
N1	134 (53.6)	91 (40.1)	117 (47.2)	103 (67.3)	43 (51.8)
* P*	0.128	0.137	0.073	0.221	0.311
**AJCC7 stage**					
I/IIA	71 (62.8)	32 (28.8)	47 (40.9)	57 (72.2)	26 (68.4)
IIB/III	231 (55.5)	153 (38.9)	185 (43.8)	188 (70.4)	77 (52.7)
* P*	0.164	0.051	0.569	0.765	0.083

Abbreviations: pT, pathologic T stage; pN, lymph node metastases; AJCC7, American Joint Committee on Cancer (Seventh Edition); PI3K, phosphatidylinositol 3-kinases;

EGFR, epidermal growth factor receptor; TIMP1, TIMP metallopeptidase inhibitor 1.

### Prognostic significance of PI3K-p85α, TIMP1, c-KIT, EGFR and p53

For analyzing the prognosis relevance, we reviewed the follow-up information of the patients. We unexpectedly found that the cases from Henan were mostly loss to follow-up. In view of such situation, we merged the available cases with follow-up information (267 of Beijing and 35 of Henan). We divided them into two cohorts: the first was of 175 cases a decade ago, and the second of 147 cases from 2006 to 2009. Clinical characteristics of patients from the two cohorts were summarized in **Table 2**.

**Table 2:** Clinicopathologic characteristics of patients with esophageal squamous cell carcinoma.

	First cohort		Second cohort		Total	
Clinical features	(n = 213)		(n = 377)		(n = 590)	
	No.	%	No.	%	No.	%
**Age at surgery, years**						
Median	61		60		61	
Range	34–78		38–88		34–88	
**Sex**						
Female	53	24.9	108	28.6	161	27.3
Male	160	75.1	269	71.4	429	72.7
**Macroscopic types**						
Medullary	130	61.0	107	41.0	237	50.0
Ulcerative	12	5.6	117	44.8	129	27.2
Fungating	66	31.0	32	12.3	98	20.7
Others	5	2.3	5	1.9	10	2.1
**Tumor location**						
Upper	24	11.4	49	13.1	73	12.5
Middle	127	60.2	235	62.8	362	61.9
Lower	60	28.4	90	24.1	150	25.6
**Tumor size, cm**						
≤5	104	50.5	198	54.0	302	52.7
>5	102	49.5	169	46.0	271	47.3
**Histology grade**						
Good (G1)	53	25.4	99	26.4	152	26.0
Moderate (G2)	116	55.5	216	57.6	332	56.8
Poor (G3)	40	19.1	60	16.0	100	17.1
**pT**						
T1/T2	20	9.4	253	67.1	273	46.3
T3/T4	193	90.6	124	32.9	317	53.7
**pN**						
N0	100	46.9	218	57.8	318	53.9
N1	113	53.1	159	42.4	272	46.1
**AJCC7 stage**						
I/IIA	34	16.0	94	25.1	128	21.8
IIB/III	179	84.0	280	74.9	459	78.2
**Follow-up time, months**						
Median	25.8		39		34.1	
Range	1–168		1–73		1–168	

Note. sums of numbers may not be added to total number of patients in cohort because of missing data. Abbreviations: pT, pathologic T stage; pN, lymph node metastases; AJCC7, American Joint Committee on Cancer (Seventh Edition).

**Figure 3:**
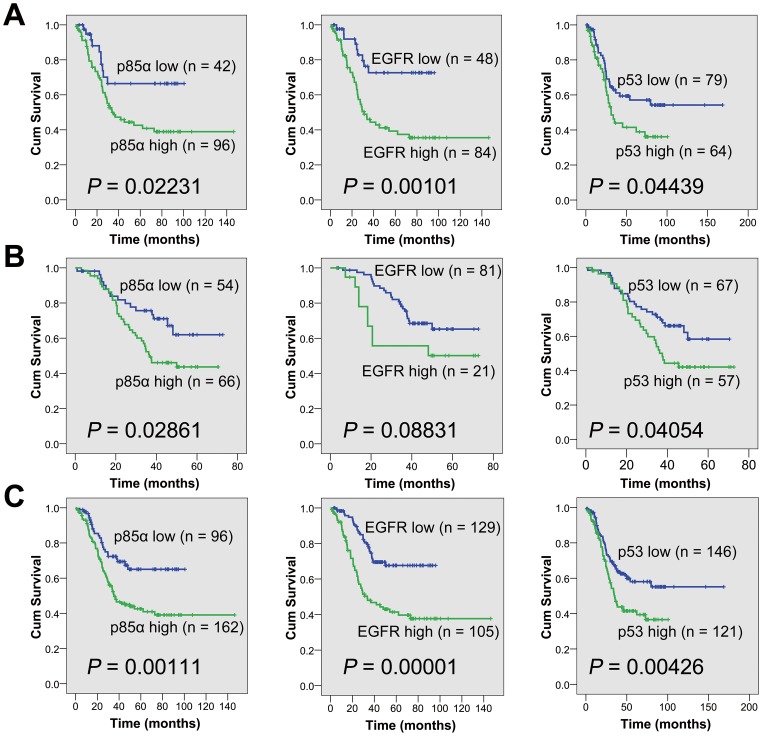
Overall survival analysis according to the expression of PI3K-p85α, EGFR and p53. (**A**) Overall survival analysis in the first cohort of 213 ESCCs. (**B**) Overall survival analysis in the second cohort of 377 ESCCs. (**C**) Overall survival analysis in a total of 590 ESCCs. Blue graph: patients with “PI3K-p85α low” or “EGFR low” or “p53 low”. Green graph: patients with “PI3K-p85α high” or “EGFR high” or “p53 high”. PI3K, phosphatidylinositol 3-kinases; EGFR, epidermal growth factor receptor.

**Figure 4:**
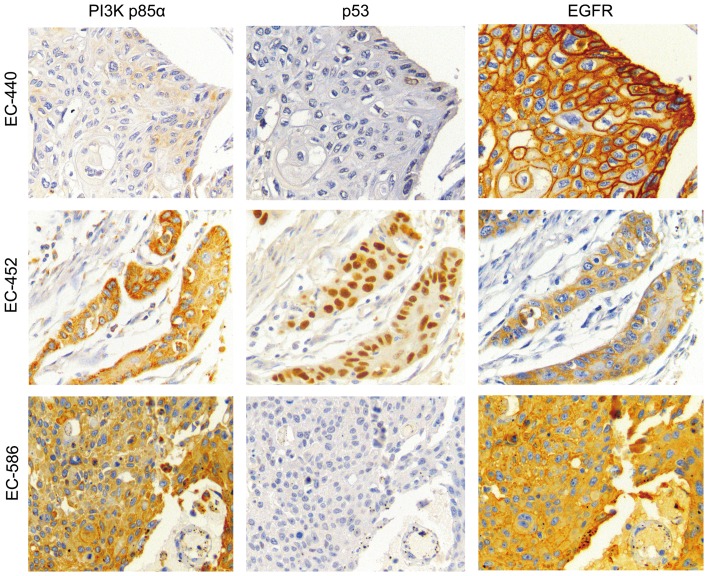
Representative IHC images of PI3K-P85α, EGFR, and p53 in the serial tissue sections. Expression of these proteins in 3 cases (EC-440, EC-452, EC-586). PI3K, phosphatidylinositol 3-kinases; EGFR, epidermal growth factor receptor. Original magnification: 400×.

**Figure 5:**
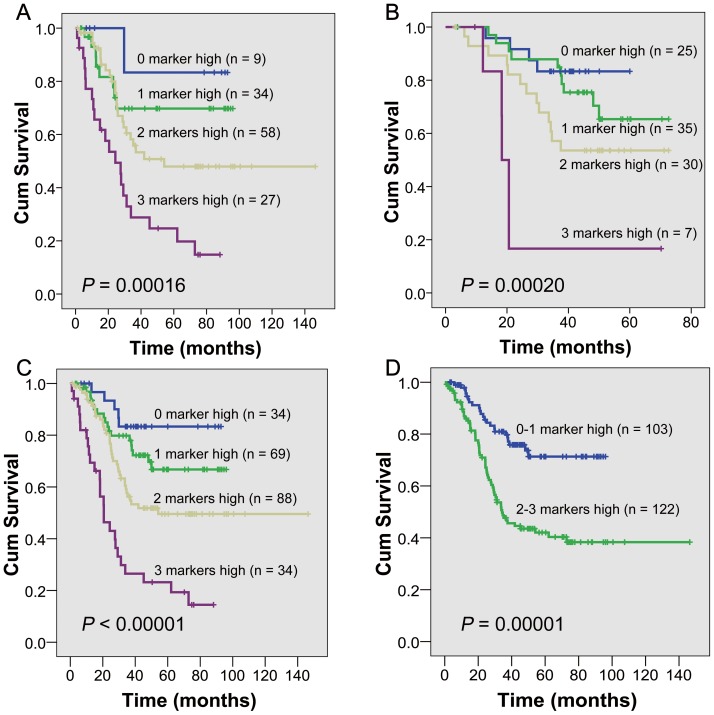
Overall survival analysis according to numbers of highly expressed proteins in ESCC tumors. (**A–C**) ESCCs are divided into four groups in both the two cohorts or together: better prognosis (high expression of 0 marker), good prognosis (high expression of 1 marker), average prognosis (high expression of 2 markers) and poor prognosis (high expression of 3 markers). (**D**) ESCCs are divided into two groups: good prognosis (high expression of 0–1 marker) and poor prognosis (high expression of 2–3 markers).

In the first cohort, high expression of PI3K-p85α (*P* = 0.02231), EGFR (*P* = 0.00101) and p53 (*P* = 0.04439) were associated with poor survivals in ESCCs, whereas no correlation was found between the abnormalities of TIMP1 or c-KIT and prognosis (**Figure 3A**). In the second cohort, high expression of PI3K-p85α and p53 also contributed to a poorer survival (*P* = 0.02861, 0.04054), whereas overexpression of EGFR (*P* = 0.08831) was not significantly correlated with a shorter overall survival (**Figure 3B**). Based on the consistency of Kaplan-Meier plots of patients with ESCC in the two cohorts, the clinical data from the two groups of samples were combined into a single database to test prognostic value of PI3K-p85α, EGFR and p53. There was a significant correlation between high expression of PI3K-p85α, EGFR and p53 and the overall survival (*P* = 0.00111, 0.00001, 0.00426, **Figure 3C**). Stratified analysis indicated that high expression of p53 was correlated with short overall survival in pN0 (*P* = 0.010) and stage I/IIA (*P* = 0.005), EGFR in pN0 (*P* = 0.003), pN1 (P = 0.002) and stage IIB/III (*P* = 0.00005) and PI3K-p85α in pN1 (*P* = 0.00007) and stage IIB/III (*P* = 0.001). Representative immunohistochemical images of PI3K-P85α, EGFR and p53 expressions in the same regions were shown in **Figure 4**. Especially, the three-protein panel (PI3K-P85α, EGFR and p53) could divide the patients into subgroups with different prognosis in both the two cohorts or together (*P* = 0.00016, 0.00020, <0.00001, **Figure 5A–C**). Patients with high expression of two or three proteins had a much poorer prognosis compared with those with zero or one high marker (*P* = 0.00001, **Figure 5D**).

### Independent prognostic value of the proteins PI3K-p85α, EGFR and p53

**Figure 6:**
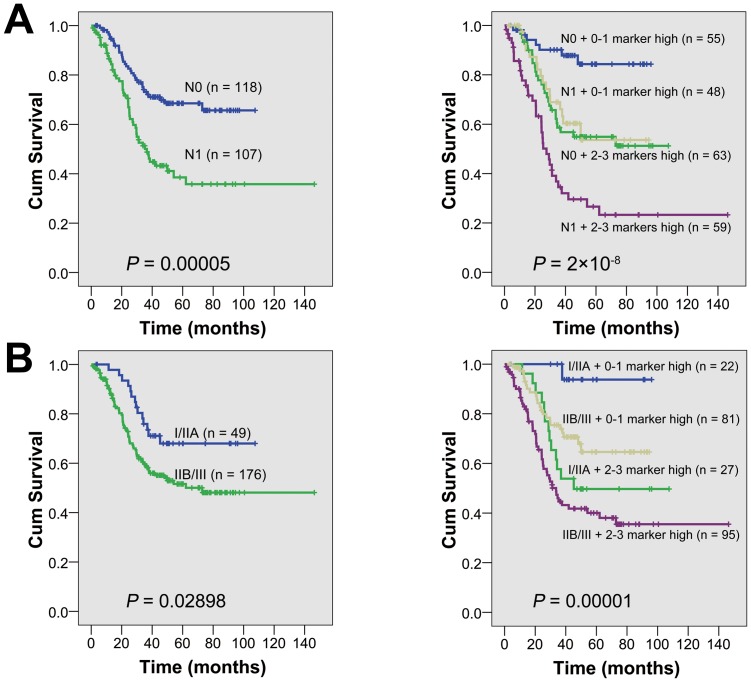
Overall survival analysis according to the combination of the protein panel and clinicopathologic parameters. (**A**) A combination of the protein panel and lymph node metastases could stratify patients more accurately (right Kaplan-Meier curves) than just only lymph node metastases (left Kaplan-Meier curves). (**B**) A combination of the protein panel and pathologic stage could stratify patients more accurately (right Kaplan-Meier curves) than just only stage (left Kaplan-Meier curves).

Based on that the three proteins (PI3K-p85α, EGFR and p53) had a significant prognostic value, we further determined by Multivariate cox regression analysis whether they could provide additional prognostic information independent of clinicopathologic features. As summarized in **Table 3**, each of them was of independent prognostic significance (*P* = 0.00003; 0.00001; 0.02293), and both PI3K-p85α and EGFR had greater prognostic values for the panel (HR: 3.688; 95% CI: 2.057–6.611; *P* = 0.00001; HR: 2.351; 95% CI: 1.466–3.769; *P* = 0.00039) than p53 (HR: 1.424; 95% CI: 0.904–2.243; *P* = 0.12740). And the three-protein panel showed more significant as an independent prognostic factor (HR = 2.090, 95% CI: 1.621–2.696, *P* = 1×10^−8^). Compared with only lymph node metastasis (pN) or pathologic stage (AJCC7), the combination of the panel and pN or pathologic stage could stratify patients more accurately (*P* = 2×10^−8^, 0.00001) (**Figure 6**).

**Table 3:** Multivariate cox regression analysis of factors predicting survival time of patients with esophageal squamous cell carcinoma.

Variable	HR	95% CI	*P*
**Model A**			
Sex (Male *VS* Female)	1.423	0.888–2.279	0.14258
Tumor size (>5 *VS* ≤5)	1.371	0.933–2.017	0.10808
Grade (G3 *VS* G1/G2)	1.226	0.771–1.950	0.38848
pT (T3/T4 *VS* T1/T2)	0.852	0.530–1.368	0.50719
pN (N1 *VS* N0)	3.265	2.170–4.912	1×10^−8^
AJCC7 stage (IIB/III *VS* I/IIA)	1.239	0.631–2.431	0.53359
PI3K-P85α (high *VS* -)	2.648	1.678–4.179	0.00003
**Model B**			
Sex (Male *VS* Female)	1.273	0.753–2.151	0.36764
Tumor size (> 5 *VS* ≤ 5)	1.436	0.933–2.208	0.09970
Grade (G3 *VS* G1/G2)	1.399	0.828–2.367	0.20992
pT (T3/T4 *VS* T1/T2)	0.902	0.539–1.508	0.69348
pN (N1 *VS* N0)	2.566	1.653–3.984	0.00003
AJCC7 stage (IIB/III *VS* I/IIA)	1.170	0.572–2.395	0.66756
EGFR (high *VS* -)	2.652	1.708–4.118	0.00001
**Model C**			
Sex (Male *VS* Female)	1.307	0.829–2.059	0.24901
Tumor size (> 5 *VS* ≤ 5)	1.282	0.877–1.872	0.19931
Grade (G3 *VS* G1/G2)	1.000	0.625–1.600	0.99919
pT (T3/T4 *VS* T1/T2)	0.936	0.580–1.510	0.78587
pN (N1 *VS* N0)	2.498	1.063–2.277	0.02293
AJCC7 stage (IIB/III *VS* I/IIA)	1.050	0.548–2.011	0.88421
p53 (high *VS* -)	1.556	1.063–2.277	0.02293
**Model D**			
Sex (Male *VS* Female)	1.463	0.853–2.507	0.16651
Tumor size (> 5 *VS* ≤ 5)	1.472	0.943–2.297	0.08889
Grade (G3 *VS* G1/G2)	1.710	1.004–2.913	0.04827
pT (T3/T4 *VS* T1/T2)	0.814	0.482–1.377	0.44389
pN (N1 *VS* N0)	3.059	1.924–4.863	2×10^−6^
AJCC7 stage (IIB/III *VS* I/IIA)	1.185	0.568–2.476	0.65084
PI3K-P85α(high *VS* -)	3.688	2.057–6.611	0.00001
EGFR (high *VS* -)	2.351	1.466–3.769	0.00039
p53 (high *VS* -)	1.424	0.904–2.243	0.12740
**Model E**			
Sex (Male *VS* Female)	1.558	0.911–2.665	0.10516
Tumor size (> 5 *VS* ≤ 5)	1.557	0.998–2.428	0.05114
Grade (G3 *VS* G1/G2)	1.533	0.911–2.580	0.10793
pT (T3/T4 *VS* T1/T2)	0.804	0.479–1.350	0.40900
pN (N1 *VS* N0)	2.459	1.558–3.882	0.00011
AJCC7 stage (IIB/III *VS* I/IIA)	1.096	0.527–2.282	0.80578
IHC panel (2–3 markers high *VS* 0–1 marker high)	2.090	1.621–2.696	1×10^−8^

Abbreviations: HR: hazard ratio; CI: confidence interval; PI3K, phosphatidylinositol 3-kinases; EGFR, epidermal growth factor receptor; pT, pathologic T stage; pN, lymph node metastases; AJCC7, American Joint Committee on Cancer (Seventh Edition); IHC, immunohistochemistry.

## Discussion

This study identifies a three-protein panel (PI3K-p85α/EGFR/p53) for the prognosis of ESCC patients, which could serve as an adjunct to current staging systems.

Previous investigations by Boone et al. and Fan et al. showed that positive expression of c-KIT was detected in 10% (10/101) and 29.9% (47/157) of ESCC tumors, respectively [16], [17]. In the present study, overexpression of c-KIT was observed in 55.9% (104/186) of ESCC tumors, but not significantly correlated with poor survival of ESCC patients. Therefore, it was not included in the prognostic panel. Akagi et al. reported that positive immunoreaction for PI3K-p110α was detectable in 50.0% (33/66) of ESCC tissues [9], while our data revealed no statistical significance of PI3K-p110α overexpression between ESCC and the adjacent normal tissues. Differences between our IHC results of c-KIT and PI3K-p110α and previously published reports may be led by different sources and clones of the antibodies, antigen retrieval methods, incubation time, and the detection system. Sharma et al. found that increased expression of TIMP1 was observed in 78% (51/65) of ESCC [21]. In our study, overexpression of TIMP1 was seen in 70.7% (246/348), of tumors, although normal operative margin tissues also presented relatively high positive rate of immunostaining (16.4%, 41/250).

p53 [22] and EGFR [6] have been previously identified as prognostic factors in ESCCs. In the present study, we confirmed the prognostic values of these two proteins. Additionally, we found that overexpression of EGFR was present more frequently in T3/T4 than T1/T2 (64.2% VS. 21.4%, *P* = 2×10^−7^). A meta-analysis of 1497 cases reported by Zhang et al. indicated that wild-type form of p53 status (low expression of p53 protein and/or wild-type *TP53* gene) was associated with high response to chemotherapy-based treatment in esophageal cancer. In our study, we did not find the correlation between p53 expression and the response to postoperative chemoradiotherapy (data not shown). This may be due to relatively small sample size of our cases with chemoradiotherapy-based treatment, to which further investigation should be addressed [23]. It has been documented that overexpression of PI3K-p85α had a close relation to the clinic stage in the progression of colorectal cancer [11]. Our data indicated that PI3K-p85α is a prognostic factor for ESCCs, which is the first report concerning PI3K-p85α alteration in ESCC tissues. In view that high expression of PI3K-p85α, EGFR and p53 were frequently detected in ESCC tumors but rarely in the adjacent normal esophageal tissues and the three-protein panel (PI3K-p85α/EGFR/p53) may be potentially applied to preoperative biopsies to provide the complementary basis for the diagnosis of ESCC.

A panel of three proteins (EGFR, TRIM44, and SIRT2) had been shown to determine prognosis for esophageal adenocarcinoma (EAC), and a combination of this panel and clinicopathologic features could stratify patients into subgroups with different prognosis [24]–[26]. In the present study, we identified a three-protein prognostic panel (PI3K-p85α, EGFR and p53) independent of clinicopathologic features. More importantly, a combination of the three-protein panel with pN or pathologic stage (AJCC7) could more significantly divide patients into distinct prognostic subgroups (*P* = 2×10^−8^, 0.00001), which may be beneficial to clinical intervention to prolong the life time of patients.

In conclusion, our data reveal that a three-protein panel (PI3K-p85α/EGFR/p53) could provide prognostic information in ESCCs independently of clinical prognostic parameters, and it may have clinical application prospect in the future.

## Supporting Information

Figure S1 **Study profile.** ESCC, esophageal squamous cell carcinoma TMA, tissue microarray array; IHC, immunohistochemistry.
(TIF)
