# Supplementary figures and images for: A Panel of Overexpressed Proteins for Prognosis in Esophageal Squamous Cell Carcinoma
Source: PLoS One. 2014 Oct 22;9(10):e111045. doi: 10.1371/journal.pone.0111045 (PMC4206450; doi:10.1371/journal.pone.0111045)

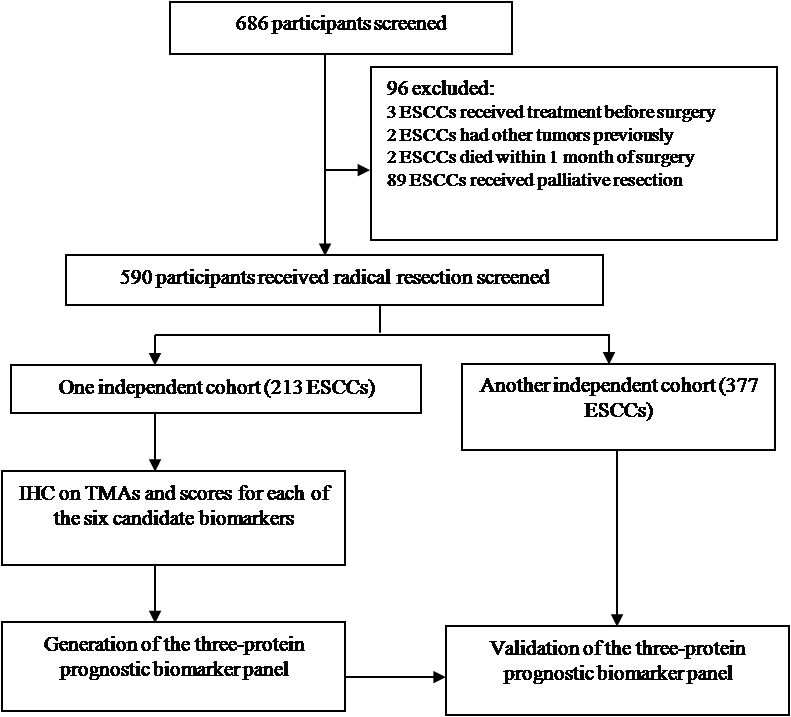

Supplement: Figure S1 — Study profile. ESCC, esophageal squamous cell carcinoma TMA, tissue microarray array; IHC, immunohistochemistry. (TIF) [file pone.0111045.s001.tif]
